# ACTIVITIES OF DAILY LIVING (ADL) NEGLECT IN INDIVIDUALS WITH DEMENTIA

**DOI:** 10.1093/geroni/igad104.0451

**Published:** 2023-12-21

**Authors:** David Hancock, Sara Czaja

**Affiliations:** Weill Cornell Medicine, New York City, New York, United States; Weill Cornell Medicine, New York City, New York, United States

## Abstract

Caregiver (CG) neglect, a form of elder mistreatment, is often measured by examining unmet care needs. A common way to measure unmet needs is to examine if a care recipient has needs for Activities of Daily Living (ADL) or Instrumental Activities of Daily Living (IADL) care needs, and if so, whether those needs are being met. The current study, using data from the Caring for the Caregiver Network Study, describes unmet ADL care needs among persons with dementia. Specifically, we examine how many needs there are, which needs are most common, how bothersome the caregiver finds attending to those needs, and how often those needs go unmet by the caregiver or other family members or friends. Overall, 1 in 10 care recipients experienced neglect as defined by unmet ADL needs. On average, caregivers (n=244) indicated care recipients needed help with 4 ADL care needs (range 0-7). The most common ADL need was needed help bathing, followed by getting dressed, and toileting. Moreover, the CGs generally found these tasks to bother or upset them between “not at all” and “a little.” Toileting and bathing were ranked as the most bothersome tasks. Additionally, 10% of the caregivers identified ADL needs which were not being met by either themselves or other family members or friends. The most common unmet need was help with bathing, followed by toileting. Needs were most bothersome and most likely unmet for intimate acts such as bathing and toileting, suggesting possible targets for skill-based intervention development.

